# Plan S – what is its meaning for open access journals and for the JACMP?

**DOI:** 10.1002/acm2.12562

**Published:** 2019-03-06

**Authors:** Michael Mills

The January, 2019 issue marked two milestones for the JACMP. It was our first monthly issue, and with it, the JACMP has published over 2000 (actually, now 2035) peer‐reviewed clinical, education, management and other science articles. The December issue of this year will mark the completion of 20 continuous years of publication. Thank you to all of our volunteers and partners for helping the JACMP reach these signposts of success.

When the JACMP was founded, some of us, including me, naïvely thought it would not be long before many if not most scholarly journals published open access. While slow, steady progress is being made in the marketplace on this front, traditional print publications, some with hybrid open access models, have proven quite resilient. Now, however, there are new developments that suggest that the traditional print model might be seeing a significant challenge.

Plan S is an initiative for open‐access science publishing that was launched by Science Europe on September 4, 2018. https://sparcopen.org/news/2018/coalition-european-funders-announces-plan-s/ It is an initiative of “cOAlition S”, https://www.coalition-s.org/, a consortium launched by the European Research Council, Science Europe, and major national research agencies and funders from twelve European countries. The plan requires scientists and researchers who benefit from state‐funded research organizations and institutions to publish their work in open repositories or in journals that are available to all by 2020. https://www.economist.com/science-and-technology/2018/09/15/european-countries-demand-that-publicly-funded-research-be-free.

Plan S makes the following assertion: “After 1 January 2020 scientific publications on the results from research funded by public grants provided by national and European research councils and funding bodies, must be published in compliant Open Access Journals or on compliant Open Access Platforms.

IN ADDITION:

- Authors retain copyright of their publication with no restrictions. All publications must be published under an open license, preferably the Creative Commons Attribution License CC BY. In all cases, the license applied should fulfil the requirements defined by the Berlin Declaration https://openaccess.mpg.de/Berlin-Declaration;
- The Funders will ensure jointly the establishment of robust criteria and requirements for the services that compliant high‐quality Open Access journals and Open Access platforms must provide;
- In case such high quality Open Access journals or platforms do not yet exist, the Funders will, in a coordinated way, provide incentives to establish and support them when appropriate; support will also be provided for Open Access infrastructures where necessary;
- Where applicable, Open Access publication fees are covered by the Funders or universities, not by individual researchers; it is acknowledged that all scientists should be able to publish their work Open Access even if their institutions have limited means;
- When Open Access publication fees are applied, their funding is standardized and capped (across Europe);
- The Funders will ask universities, research organizations, and libraries to align their policies and strategies, notably to ensure transparency;
- The above principles shall apply to all types of scholarly publications, but it is understood that the timeline to achieve Open Access for monographs and books may be longer than 1 January 2020;
- The importance of open archives and repositories for hosting research outputs is acknowledged because of their long‐term archiving function and their potential for editorial innovation;
- The “hybrid” model of publishing is not compliant with the above principles;
- The Funders will monitor compliance and sanction non‐compliance. https://www.scienceeurope.org/wp-content/uploads/2018/09/Plan_S.pdf

First, note this is no small initiative with only a few supporters. The coalition represents 13 of the 43 major national research funding organizations in Europe and 2 European charitable foundations, including:

- Der Wissenschaftsfonds
- Academy of Finland
- Agence Nationale de la Recherche
- Science Foundation Ireland
- Institute Nazionale di Fisica Nucleare
- Luxembourg National Research Fund
- Netherlands Organisation for Scientific Research
- The Research Council of Norway
- National Science Centre Poland
- Slovenian Research Agency
- Swedish Research Council for Health, Working Life and Welfare
- FORMAS
- UK Research and Innovation
- Wellcome
- Bill & Melinda Gates Foundation
- Riksbankens Jubileumsfond — The Swedish Foundation for Humanities and Social Sciences
- European Commission Science Council
- Science Europe

Supporting statements for this initiative from many of the above and others can be found here: https://www.coalition-s.org/funders-and-supporters/. The Coalition plans to work with the Directory of Open Access Journals (DOAJ) to identify and assure the journals it credentials meet its requirements as completely open access. https://www.coalition-s.org/feedback/, https://doaj.org/.

The JACMP is listed in the DOAJ along with over 12,500 other open access journals. It seems unlikely that most funded research would not be able to identify an appropriate journal for its subject matter. The JACMP's DOAJ listing follows:

Journal of Applied Clinical Medical Physics
ISSN: 1526‐9914 (Online)
http://onlinelibrary.wiley.com/journal/10.1002/(ISSN)1526-9914
Double blind peer review
Subject: Medicine: Medicine (General): Medical physics. Medical radiology. Nuclear medicine
Date added to DOAJ: 5 Dec 2017
Record Last Updated: 5 Dec 2017

Open Access journals and platforms need to meet the following criteria to be compliant with Plan S:

- All scholarly content must be immediately accessible upon publication without any delay and free to read and download, without any kind of technical or other form of obstacles.
- Content needs to be published under CC BY, CC BY‐SA or CC0.
- The journal/platform must implement and document a solid review system according to the standards within the discipline, and according to the standards of the Committee on Publication Ethics.
- The journal/platform must be listed in the DOAJ or be in the state of being registered.
- Automatic APC waivers for authors from low‐income countries and discounts for authors from middle‐income countries must be provided.
- Details about publishing costs (including direct costs, indirect costs, and potential surplus) impacting the publication fees must be made transparent and be openly available on the journal website/publishing platform.
- DOIs must be used as permanent identifiers.
- Long‐term digital preservation strategy by deposition of content in an archiving programme such as LOCKSS/CLOCKSS.
- Accessibility of the full text in a machine‐readable format (e.g., XML/JATS) to foster text and data mining.
- Link to raw data and code in external repositories.
- Provide high quality and machine‐readable article level metadata and cited references under a CC0 public domain dedication.
- Embed machine readable information on the Open Access status and the license of the article.

The AAPM and Wiley are working to ensure that the JACMP will be compliant with all the particulars of Plan S and therefore be eligible as a Coalition approved journal. https://en.wikipedia.org/wiki/Plan_S

Not surprisingly, publishers have given Plan S a frosty reception. The policy “potentially undermines the whole research publishing system,” said Springer Nature, which publishes more than 3,000 journals, including Nature. The American Association for the Advancement of Science (AAAS), which publishes Science, said it would “disrupt scholarly communications, be a disservice to researchers, and impinge academic freedom”. https://www.economist.com/science-and-technology/2018/09/15/european-countries-demand-that-publicly-funded-research-be-free

More than 1,800 academics have signed an open letter that voices support for open‐access publishing mandates from funders. Although the letter, which was spearheaded by Eisen, does not directly reference Plan S, it states that while funder demands may “superficially limit our publishing option in the short term,” they can lead to a system that “[maximizes] the reach of our scholarship and its value to the research community and public.” http://michaeleisen.org/petition/index.php

In early November, more than 600 researchers signed a different open letter — this one criticizing the plan for being “unfair for scientists” and “too risky for science in general.” The letter states that Plan S is a “serious violation of academic freedom,” and outlined several specific problems the academics have with the plan, including a ban on many valuable journals, the possible risk to international collaboration if funders in others parts of the world did not adopt a similar policy, and the potential for the cost of scholarly dissemination to increase under a model focused on “gold” open access, in which authors pay article processing charges (APCs) — sometimes in the thousands of dollars — for individual papers. https://zenodo.org/record/1477914#.XBdtOhNKhTa, https://www.the-scientist.com/news-opinion/plan-s-the-ambitious-initiative-to-end-the-reign-of-paywalls-65231.

So far, 16 funders, most of them in Europe, have embraced Plan S, not enough to transform journal finances. U.S. government funders remain cool to the approach. But Plan S's international momentum grew — along with the threat it poses to traditional publishing — in December 2018, when officials in China backed its open‐access goals. If China follows through, Plan S could reduce publishers' income by perhaps 15% under certain conditions, according to an estimate published last week by Delta Think, a consulting firm in Philadelphia, Pennsylvania. That analysis does not include the effect of the cap on author fees (also called article‐processing charges), which could cut revenues further. The average fee for papers published in purely open‐access journals in 2018 was about $1600, Delta Think has estimated. https://www.sciencemag.org/news/2019/01/scientific-societies-worry-plan-s-will-make-them-shutter-journals-slash-services

U.S. federal agencies are sticking to policies developed after a 2013 White House order to make peer‐reviewed papers on work they funded freely available within 12 months of publication. “We don't anticipate making any changes to our model,” said Brian Hitson of the U.S. Department of Energy in Oak Ridge, Tennessee, who directs that agency's public access policy.

Nor are the three main federal research funders in Canada ready to change their joint 2015 OA policy. Plan S is “a bold and aggressive approach, which is why we want to make sure we've done our homework to ensure it would have the best effect on Canadian science,” says Kevin Fitzgibbons, executive director of corporate planning and policy at Canada's Natural Sciences and Engineering Research Council in Ottawa. https://www.sciencemag.org/news/2019/01/will-world-embrace-plan-s-radical-proposal-mandate-open-access-science-papers

But a number of commentators on the plan, from both the publishing and academic communities, have raised the possibility of collateral damage to another big part of the scholarly ecosystem: nonprofit societies. These nonprofits fund a large share of their activities in support of their communities through subscription revenues from journal publishing. And some critics assert that, ironically, Plan S could well drive some of them out of the publishing business entirely, further concentrating power in the hands of the big commercial players. https://www.osa-opn.org/home/newsroom/2018/December/europe_s_plan_s_casts_shadow_on_scholarly_societie/

In my view, this would seem to be the most likely eventuality: I believe the goals of Plan S could eventually be realized, perhaps substantially by 2025, and almost completely by 2030. It might be that the revenue currently enjoyed by the large publishers and scientific societies, could be reduced by 15% or more. This is just a guess, but a much more informed one than 20 years ago. Plan S could eventually succeed, and if it does, it may result in a publication model that better serves the public interest. For the JACMP, the next years could result in continued growth, particularly in Europe and China, where there is anticipated to be significant momentum to publish in Gold open access journals that are compliant with Plan S and the goals of cOAlition S.

Finally, I want to remind everyone that the JACMP and Medical Physics Journal app can be found on the AAPM Publications page:https://www.aapm.org/pubs/ and on the Medical Physics Journal page http://www.medphys.org/

